# Intimations of a New Paradigm for Lexical Studies in Psychology

**DOI:** 10.3389/fpsyg.2022.867491

**Published:** 2022-07-01

**Authors:** Gerard Saucier

**Affiliations:** Department of Psychology, University of Oregon, Eugene, OR, United States

**Keywords:** personality structure, individual differences, factor structure, culture, language

## Introduction

Several consecutive productive decades of research on basic dimensions of personality have given a firmer footing to the discipline due, in part, to contributions of lexical studies of personality. These studies helped establish more consensus as to structure—a set of a few dimensions worth measuring—which has enabled knowledge to accumulate within something of a standard assessment paradigm. Admittedly, these dimensions are quite broad, and if one wishes to characterize their content quite accurately, he or she must often resort to compound labels (e.g., honesty/humility, intellect/imagination). The factors they refer to are, basically, collages of more fine-grained components; the composition of the collages varies somewhat from one measure to another, and one language to another.

There are, however, cracks in the paradigm. The “few dimensions” most widely regarded as measurement worthy—the Big Five—embody a good degree of ethnocentric bias, deriving from origin in Western samples and studies in and of Germanic-language-family contexts. These dimensions often replicate only partially or weakly. And, although these have good evidence for comparative predictive validity (in competition with SES and IQ; Roberts et al., 2007), the magnitude of prediction is not overwhelming. Better prediction is likely to come from more comprehensive models (Paunonen and Ashton, 2001; Mõttus et al., 2017). In lexical studies, five factors account for only a narrow slice—about a quarter—of the variance that stimuli based on the personality vocabulary can afford (e.g., Saucier and Iurino, 2020). Personality data are not 75% noise! Structures with far more than five factors (accounting for closer to half the variance) have recently been found to be replicable to a good degree (Saucier and Iurino, 2020).

## The Comparative Value of More Fine-Grained Personality Dimensions

From the standpoint of cultural psychology, variables/constructs might be located on a spectrum from universal to culture specific, with gradations of generality between, the collage-of-content broad personality factors (e.g., Big Five) are difficult to place on this spectrum, because the make-up of the collage (e.g., that for agreeableness) differs somewhat by language/cultural context, obscuring which subcomponents are more vs. less universal. The field of emotion (also, to a degree, psychopathology) does not have this problem, because of a tradition of a long practice of studying many fine-grained constructs and, thereby, concluding, for example, that anger is a more universal concept than *schadenfreude*.

Where does one locate useful fine-grained constructs for personality? Saucier et al. (2014) compared trait categories based on single words across 12 relatively remote languages *via* English translation, leading to insights into what person-descriptive concepts might be most universal. In essence, they used the full compendium of dictionary-derived terms in a language as a background matrix for comparison. This imposingly complex different background framework could be improved by grouping synonyms into clusters (as in Wood et al., 2020; also Goldberg, 1990); this is difficult to do without much arbitrariness and subjectivity, but, perhaps, a distributional-semantics approach could eventually accomplish it.

Factor analysis might yield a more tractable background matrix for cross-cultural comparisons, since recent studies (e.g., Saucier and Iurino, 2020) have indicated structures with 30 or more independent factors with relatively fine-grained content can show much across-method robustness within a large set of study data. There could be much content overlap between components of such highly differentiated, high-dimensionality structures derived from different languages: some kinds of content arising ubiquitously (possible universals), other kinds potentially more unique to one language, and other kinds on the spectrum in-between. Besides their value in settling basic-research questions about universality-vs.-culture specificity of constructs, what emerges from this comparative research on fine-grained personality factors could be high-dimensionality models, predicting outcomes well more powerfully than current five- (or six-) factor models, and more powerfully than structures of so-called facets of those factors (as identified in questionnaires), because facets are markedly more highly correlated (i.e., partially redundant) than factors in high-dimensionality structures.

A high-dimensionality approach does not require discarding of low-dimensionality models, which will be found nested (in some way) in the high-dimensionality structure. Few-factor models are more parsimonious, *may*, generally, be at least, slightly more replicable, and are more tractable for theory development. Complementarily, high-dimensionality models are more comprehensive and should, in aggregate, predict outcomes markedly better. The trade-offs involved need routine attention.

## Optimal Method Characteristics

What are optimal method characteristics for such research? First, some culture fairness is in order. Personality-structure research over the last 40 years has emphasized comparisons to a background structure of five or six dimensions, structures based almost entirely on studies of European languages (Hofstee et al., 1997; Ashton et al., 2004). If such research had originated in multiple languages of Asia or Africa, it would have almost certainly had a different start point. A background-matrix model would be better derived from systematic comparison of well-conducted studies in several widely diverse (geographically, culturally) languages. But there are other critiques.

The typical features of a lexical study within a single language have come to include the following: reduction of a large set of potential individual-differences terms down to only a few hundred on which large data are gathered, privileging self over peer data, ipsatizing data and ignoring results that might arise from the original data, determining the number of factors by an informal visual-scree test, with little attention to comparative stability/replicability of structures, assuming that proper structure is in the vicinity of five factors rather than at far more differentiated levels (that account for much more variance in the data), and using a particular factor-rotation method that is ill-equipped to capture high-dimensionality structure. Before defining a more preferable method package (that would enable identification of both broad, collage factors and more fine-grained ones), I will critique each of the aforementioned components of the current standard method package. I take these in reverse order, getting to the more fundamental aspects of study design last. Recent empirical studies (Saucier and Iurino, 2020; Saucier et al., 2020; Thalmayer et al., 2020, 2021) have demonstrated the utility of these approaches.

1. Recent lexical studies deriving high-dimensionality structures have revealed the varimax-rotation approach to constrain rather systematically the number of meaningful factors; it seems difficult to find viable varimax structures with more than about a dozen factors in lexical-study data. Varimax tends to retain multiple large factors that have a high variance in their loadings column, but not greatly break these up as more factors are rotated. Thus, it constrains the number of meaningful factors. Oblimin and equamax rotations do not have so much of this constraint and are good additions to the method arsenal. Certainly, it is good if multiple rotations lead to similar results, and that is an important criterion for robustness; Goldberg (1990) convincingly showed that one rotation vs. another produces little variation in results in English-language data at the five-factor level, but this does not mean that these rotations are *always* interchangeable, enabling one to use varimax and discard major alternatives.
2. The scree test looks for significant flattening out in the relative magnitude of eigenvalues as one extracts more (unrotated) factors. But determination of where the single most meaningful flattening occurs in the plot is somewhat subjective and, in some cases, may depend on how one stretches the graph vertically or horizontally. Parallel analysis compares the scree from current data with that from random data, indicating how many of the (unrotated) factors are larger than what would be expected in random data, a more precise determination than the scree test, often indicating more factors than visual scree would suggest. But parallel analysis may not be the optimum. Like the scree test, it focuses on the size of *unrotated* factors, whereas modern approaches to factor structure are based on the rotated versions, which have different eigenvalues (for which the sums-of-squared loadings, analogous to eigenvalues, would generate a flatter scree plot). If there were a factor structure with more factors than parallel analysis (or the scree test) would dictate, but in which all factors were interpretable, replicable, and sufficient sized (e.g., four substantial-loading items out of a 1,000-item pool) met a minimum criterion of sufficient size, why disregard it? Whether parallel analysis provides an absolute authoritative ceiling on factor number in these studies is a researchable issue. Our recent work has suggested one finds a better ceiling in structures accounting for 50–60% of variance. The general point: research on personality structure needs to get beyond a fixation—supported by the echo chamber, building up since the earliest studies—on only the first few factors that emerge.
3. Many early studies of lexical personality structure condensed the variables into bipolar scales or clusters (e.g., Goldberg, 1990, 1992) before the norm of analyzing single variables became set in the early 1990s. From then, the method package has typically included ipsatization of the data (standardizing responses for each participant, removing differences in scale usage, including acquiescence, by a brute-force approach). This response-bias control is valuable, and factors generated from ipsatized data are more likely to be truly bipolar (high loadings in both directions). But ipsatization can make for distortive results when content is confounded with keying direction or response means asymmetrically distributed (Dunlap and Cornwell, 1994; Ten Berge, 1999). And once a structure is identified, even if one sticks with lexical items, he or she typically assesses individuals thereafter, using raw/original data again. All else equal, given this back and forth and the caveats associated with each data type, it would be better if a structure was robust across variations in data type—whether the data were original or ipsatized. Canonical-correlation analysis (proportion of variance shared) provides a useful comparative tool.
4. To return to the number-of-factors issue, important criteria distinguishing alternative candidate structures involve replicability. How replicable is each candidate structure across variation in data type (original or ipsatized), rotation method (e.g., orthogonal vs. oblique), self- vs. peer ratings (if both are available), and any prominent demographic subcategories (e.g., female-male) within the sample? All else equal, a good structure should arise resiliently despite method variations.
5. The first two attempts to carry out a modern-style lexical study (in the era of the decade beginning in 1972) retained over 1,000 variables for study (Brokken, 1978; Goldberg, 1990, Study 1). But subsequent studies have retained far less, understandably, because 1,000+-variable questionnaires make for more difficult recruitment of participants. But, obviously, the number of variables analyzed has some effect on how many dimensions will be identified; Saucier and Iurino (2020) had candidate structures with more factors when based on 1,710 rather than 587 variables. Some 30 years ago, it was still problematic to analyze such a large number of variables at once, but not any longer. It would be a welcome development if the field returned to larger collections of variables. So long as the terms are all frequently used and understandable, and the participants are suitably compensated for their time investment. This would enable identification of more differentiated and more comprehensive and ultimately predictive structures.
6. Samples would ideally be similarly large (*N* > 1,000 where feasible), making correlational estimates more precise. And there is no particular reason to privilege self-over peer-rating data, especially since self-rating data are an awkward format for some traditional cultural settings (e.g., Thalmayer et al., 2020) where peer-rating data are readily obtainable. Gossip seems to be more universal than volunteering the details of one's self-concept.

## Discussion

Lexical studies of personality descriptors reached virtual stasis as a standard paradigm nearly three decades ago. But recent methodological and conceptual innovations have yielded results bespeaking a need for revisions in the paradigm. Recent studies have indicated potential for substantial advances over results obtained with the standard paradigm. The interconnected recommendations for studies made above do not provide a fully defined paradigm but set out a basic framework within which further innovations can accrue as relevant research builds up.

In conclusion, development of useful models of the high (not just low) end of personality structure will provide important advantages. Although some details of best methods for identifying high-dimensionality structures remain to be conclusively defined, much of a new paradigm is in place. Analyses of new data and re-analyses of archived data from previous projects can be harnessed to yield new insights into the natural organization of personality tendencies, without imposing current ethnocentrically biased few-factor models.

## Author Contributions

The author confirms being the sole contributor of this work and has approved it for publication.

## Conflict of Interest

The author declares that the research was conducted in the absence of any commercial or financial relationships that could be construed as a potential conflict of interest.

## Publisher's Note

All claims expressed in this article are solely those of the authors and do not necessarily represent those of their affiliated organizations, or those of the publisher, the editors and the reviewers. Any product that may be evaluated in this article, or claim that may be made by its manufacturer, is not guaranteed or endorsed by the publisher.
